# Population attributable risk of breast cancer in white women associated with immediately modifiable risk factors

**DOI:** 10.1186/1471-2407-6-170

**Published:** 2006-06-27

**Authors:** Christina A Clarke, David M Purdie, Sally L Glaser

**Affiliations:** 1 Northern California Cancer Center, Fremont, California, USA and Department of Health Research and Policy, Stanford University Medical Center, Stanford, California, USA

## Abstract

**Background:**

Estrogen/progestin replacement therapy (EPRT), alcohol consumption, physical activity, and breast-feeding duration differ from other factors associated with breast cancer in being immediately modifiable by the individual, thereby representing attractive targets for future breast cancer prevention efforts. To justify such efforts, it is vital to quantify the potential population-level impacts on breast cancer considering population variations in behavior prevalence, risk estimate, and baseline incidence.

**Methods:**

For each of these four factors, we calculated population attributable risk percents (PARs) using population-based survey (2001) and cancer registry data (1998–2002) for 41 subpopulations of white, non-Hispanic California women aged 40–79 years, and ranges of relative risk (RR) estimates from the literature.

**Results:**

Using a single RR estimate, subpopulation PARs ranged from 2.5% to 5.6% for hormone use, from 0.0% to 6.1% for recent consumption of >= 2 alcoholic drinks daily, and 4.6% to 11.0% for physical inactivity. Using a range of RR estimates, PARs were 2–11% for EPRT use, 1–20% for alcohol consumption and 2–15% for physical inactivity. Subpopulation data were unavailable for breastfeeding, but PARs using published RR estimates ranged from 2% to 11% for lifetime breastfeeding >= 31 months. Thus, of 13,019 breast cancers diagnosed annually in California, as many as 1,432 attributable to EPRT use, 2,604 attributable to alcohol consumption, 1,953 attributable to physical inactivity, and 1,432 attributable to never breastfeeding might be avoidable.

**Conclusion:**

The relatively feasible lifestyle changes of discontinuing EPRT use, reducing alcohol consumption, increasing physical activity, and lengthening breastfeeding duration could lower population breast cancer incidence substantially.

## Background

Reducing the morbidity and mortality associated with breast cancer, the most commonly diagnosed malignancy in women, is a pressing public health concern. One part of the response to the growing breast cancer burden is to develop effective prevention strategies. Although many breast cancer risk factors have been identified that might form the basis of such strategies, prevention remains challenging, due in large part to practical difficulties in modifying risk-increasing factors like nulliparity, late age at first full-term pregnancy, early age at menarche, and late age at menopause[1,2]. However, some established risk factors for breast cancer could be considered for practicable modification because they involve immediately modifiable personal behaviors of adulthood. Examples include recent use of estrogen/progestin -containing replacement therapy (EPRT)[3], recent alcohol consumption[4], shorter breastfeeding duration[5], and physical inactivity[6,7].

Implementing modifications in any of these behaviors would require considerable efforts in health education and policy. To justify such preventive strategies, it is important first to quantify the theoretical impacts of changes on breast cancer incidence at the population level, but to date few analyses of this sort have occurred[8-10]. A reasonable epidemiologic metric, the population attributable risk (PAR), exists for estimating the effect on disease rates of removing a risk factor from the population. However, since the PAR takes into consideration both disease incidence and risk factor prevalence, and since these are well-established to vary across population subgroups, a PAR determined for a single population has limited utility for understanding the broader range of impact that risk factor changes might have on breast cancer incidence.

Data from California, USA provide an as-yet unexplored opportunity to circumvent this problem, since this state has a large, demographically heterogeneous population and routinely collects detailed breast cancer incidence and risk factor data at the relatively small subpopulation level of the county. In addition, California counties demonstrate variation in both breast cancer incidence rates and socioeconomic status generally representative of national variation[11]. Therefore, we took advantage of California county-level cancer registry and risk factor surveillance data to estimate potential reductions in breast cancer incidence for changes in four modifiable behaviors–usage of EPRT, consumption of alcohol, physical inactivity and sustained breastfeeding.

## Methods

### Analytic rationale

The PAR is calculated using the population prevalence of the exposure (p) and the relative risk associated with the exposure (r), as follows: PAR = p(r-1)/(1+p(r-1))[12]. We obtained estimates of the prevalence and effect of the exposures under consideration from population risk factor surveys and published epidemiological studies, as described below. We then applied the PARs to breast cancer incidence rates for each of 41 California county-based subpopulations defined by the California Health Interview Survey (CHIS) to estimate the potential change in case counts from reductions in exposure prevalence. We limited our assessments to women of white, non-Hispanic race/ethnicity (hereafter referred to as white) in order to minimize the influence of racial/ethnic distributions on differences among subpopulations, and because the most reliable data on the risks of breast cancer associated with these exposures are from studies that included mostly white women[3,4]. Furthermore, 2000 US Census data show that Californian white women are generally similar to white women nationally on most sociodemographic characteristics but with slightly higher levels of education (21% vs.17% with bachelor's degree) and annual household income ($53K vs. $45K)[13]. Lastly, we restricted our analyses to women aged 40–79 years, who are at highest risk of breast cancer.

We estimated PAR point estimates for two circumstances: 1) for each of the 41 California subpopulations while utilizing a single value for the relative risk (RR), in order to determine the range of likely PAR values across defined subpopulations; and 2) for hypothetical populations with varying exposure prevalences and varying degrees of risk associated with those exposures, utilizing a range of likely values for the RR and a realistic range of exposure prevalences based on the observed subpopulation distributions.

### Breast cancer incidence and population data

Using data from the population-based California Cancer Registry and population estimates from the California Department of Finance, we calculated average annual incidence rates of invasive breast cancer (ICD-O-3 site codes 50.0–50.9) for the period 1998–2002 separately for each of the 41 county groups. The software program SEER*Stat. ([14] was used for all calculations.

### Risk factor prevalence data

Risk factor prevalence ranges were estimated using data collected by the CHIS program in 2001. The overall response rate to the adult survey was 38%, reflecting both the screener completion rate (i.e., success in surveying a household to select a respondent) of 59%, and the extended interview completion rate (i.e., success in interviewing the selected respondent) of 64%[15]. We used the internet-based AskCHIS[16], to obtain prevalences of EPRT use, alcohol consumption, and physical inactivity for each of the 41 county groups. To assess hormone therapy (HT) use, CHIS asked women aged 40 and older who were not pregnant: "Are you currently taking any hormone replacement supplements prescribed by a medical doctor to control the symptoms of menopause?" As this question referred to any kind of HT, to estimate more closely the population prevalence of women using EPRT we excluded from the numerator of our prevalence calculations any women who reported both HT use and hysterectomy (approximately half of all women reporting HT use), because women with intact uteri are not prescribed estrogen-only HT because of demonstrated risks of endometrial cancer[17].

To assess current alcohol consumption, CHIS asked all adults: "During the past month, have you had at least one drink of any alcoholic beverage such as beer, wine, wine coolers, or liquor?"; those answering yes were also asked: "During the past month, how many days per week or per month did you drink any alcoholic beverages, on the average?" and "On the days when you drank, about how many drinks did you drink on the average?" Answers to these questions were used to calculate the prevalence of women who reported drinking two or more alcoholic drinks per day on 10 or more days in the last month.

To assess physical inactivity, CHIS asked all adults a series of questions about their physical activity in the past 30 days, including whether respondents walked or bicycled to work/errands. The CHIS variable "level of physical activity" consolidated all of these questions into an indicator with three levels: "no vigorous/moderate activity", "some vigorous/moderate activity but doesn't walk/bicycle", and "some vigorous/moderate activity and does walk/bicycle". We used this variable to calculate the percentage of white women aged 40–79 years who reported no vigorous/moderate activity in the last month.

Information regarding breastfeeding is not available from CHIS. However, a recent review of population-based surveys of breastfeeding prevalence[18], estimated that 60–75% of parous white women initiated breastfeeding during the period 1994–2001, suggesting that the prevalence of not initiating breastfeeding ranged from 25% to 40%.

### Estimates of relative risk

We obtained estimates of RRs for each risk factor as follows:

#### EPRT

The Women's Health Initiative (WHI), the only randomized controlled trial of EPRT vs. placebo[3] found an elevated risk of invasive breast cancer in participants after a mean 5.2 years of follow-up, with a hazard ratio of 1.26 (95% confidence interval (CI) 1.00–1.59). A subsequent meta-analysis (including this trial) concluded that RRs of breast cancer among current users of estrogen/progestin ranged from 1.2–1.4 and increased with duration of use[19], compared to never users, although other studies estimated the RR for current, long-term use to be as high as 1.5[20,21]. Therefore, we used an RR of 1.26 for the county-specific PAR calculations and a range of RRs from 1.2–1.4 to estimate PARs for hypothetical populations.

#### Alcohol consumption

A pooled analysis of 53 epidemiologic studies[4] identified RRs of breast cancer associated with consumption of two or more alcoholic drinks per day (≥ 30 grams per day) as compared to no drinks in the range of 1.3–1.4. A pooled analysis of cohort studies[22] found that, compared with non-drinkers, women consuming 30–60 grams of alcohol daily had a multivariate-adjusted RR of 1.41 (95% CI 1.18–1.69). For one of the California subpopulations (Marin county), RRs associated with drinking alcohol daily were 2.3 (95% CI 1.2–4.4) for 2 glasses and 3.6 (95% CI 1.2–11.5) for 3+ glasses[23]. Therefore, we used an RR of 1.4 for the county-specific calculations and a range of 1.4–2.2 for estimating PARs for hypothetical populations.

#### Physical inactivity

A review of 36 epidemiologic studies identified RRs for breast cancer associated with inactivity compared to moderate physical activity in the range of 1.3–1.4 irrespective of menopausal status[6]. This range was further supported in a subsequent review by a National Cancer Institute working group[7]. Therefore, we used an RR of 1.3 for the county-specific calculations and a range of RRs from 1.2–1.4 for estimating PARs for hypothetical populations.

#### Breastfeeding

A pooled analysis of 47 international breast cancer studies (cohort, and hospital-based and population-based case-control)[5] found that the RR for breastfeeding among parous women decreased by 4.3% (95% CI 2.9%–5.8%) for every 12 months beyond the influence of additional births. With adjustment for parity and compared to women who never breastfed, the RR of breast cancer was 0.88 (95% CI 0.82–0.94) for women who breastfed 31–54 months and 0.73 (95% CI 0.63–0.83) for women who breastfed for 55+ months over their lifetimes. For analyses estimating PARs in hypothetical populations, we used RR estimates in the range of 1.1–1.3 for not breastfeeding compared with breastfeeding for at least 31 months.

## Results

### Subpopulation variation in breast cancer incidence

Overall, the average annual age-adjusted incidence rate of invasive breast cancer among California white women aged 40–79 years (331.6 per 100,000) was slightly higher than the comparable national rate reported by SEER (315.6 per 100,000). Table 1 presents breast cancer incidence rates for each county-group subpopulation. Counties with the highest incidence rates (the urban areas of Marin and San Francisco) had rates 45% higher than counties with the lowest rates (the rural areas of Imperial and Siskiyou/Lassen/Modoc/Trinity).

### EPRT

**Figure 1:**
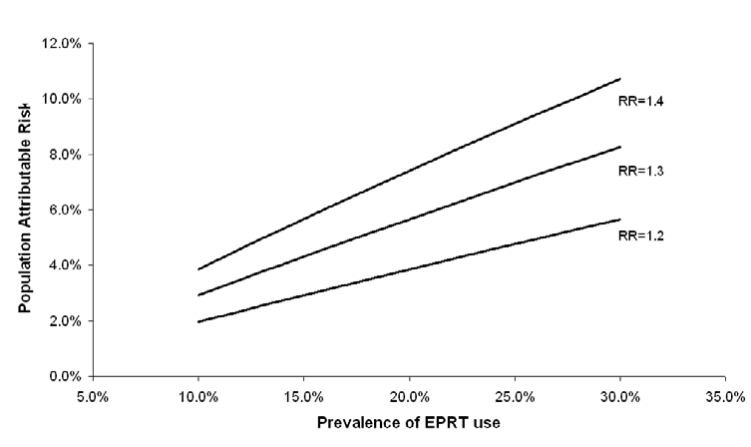
Population attributable risk of breast cancer associated with use of combined estrogen/progestin replacement therapy (EPRT) for various levels of exposure and relative risk.

In 2001, prior to the release of WHI results, EPRT use varied by over 100% among counties, ranging from 10.0% to 22.9% in a manner generally correlating with breast cancer incidence rate (Table 2). Using an RR estimate of 1.26[3], we found that the county-specific PAR estimates for current EPRT use ranged from 2.5–5.6%, (Table 2). Statewide, we estimated that approximately 4.5% of breast cancer cases (567 cases) annually could be attributed to current use of EPRT. With a range of RR estimates and population prevalences, PAR values for EPRT use ranged from 2% (assuming EPRT utilization of 10% and a relative risk of 1.2) to 11% (assuming EPRT utilization of 30% and a relative risk of 1.4) (Figure 1).

### Alcohol consumption

**Figure 2:**
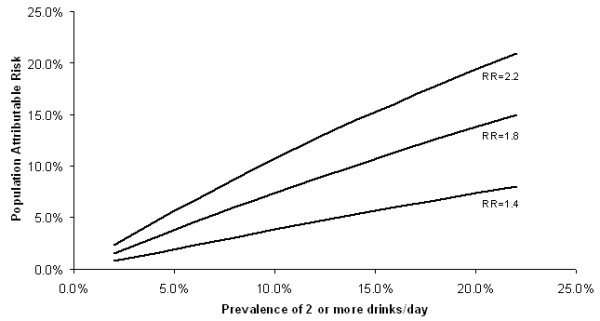
Population attributable risk of breast cancer associated with consumption of 2 or more alcoholic drinks per day for various levels of population prevalence and relative risk.

Consumption of at least two alcoholic beverages per day among white women aged 40–79 varied by over 100% among counties, ranging from 0% to 16.4% also in a manner generally correlating with breast cancer incidence rate (Table 2). Prevalence was notably higher in northern (Marin, Napa) than southern (Orange, Los Angeles) urban counties. Using an RR estimate of 1.4[22], PAR estimates for consumption of 2+ alcoholic drinks per day ranged between 0.0% and 6.1% for county groups (Table 2). Overall in California, we estimated that about 3.5% of breast cancer cases, or 450 per year were attributable to this level of alcohol consumption. Considering ranges of RR estimates from 1.4 to 2.2 and population prevalences from 2% to 22%, we produced a corresponding range of PAR values for alcohol consumption from 1% to 20% (Figure 2).

### Physical inactivity

**Figure 3:**
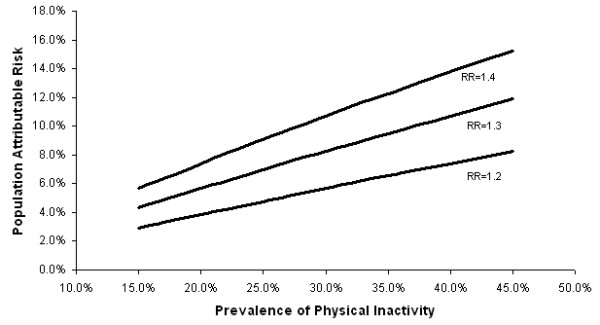
Population attributable risk of breast cancer associated with physical inactivity (reporting no moderate/vigorous activity in past 30 days) for various levels of population prevalence and relative risk.

White women aged 40–79 years reporting no vigorous/moderate physical activity in the last month ranged from 16.1 to 41.0% by county (Table 2). Using an RR estimate of 1.3[6], PAR estimates for physical inactivity ranged between 0.0% and 6.1% for county groups (Table 2). We estimated that statewide, 7.5% of breast cancer cases, or 1,422 cases per year, were attributable to a sedentary lifestyle. Considering ranges of RR estimates from 1.2 to 1.4 and population prevalences from 15% to 45%, we estimated a corresponding range of PARs of 2%–15% (Figure 3).

### Breastfeeding

**Figure 4:**
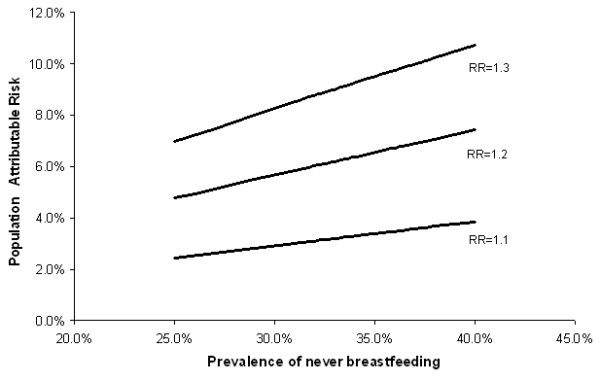
Population attributable risk of breast cancer associated with not breast feeding (compared to breastfeeding for 31 months or more over a lifetime) for various levels of population prevalence and relative risk.

Using prevalences of never breastfeeding ranging from 25% to 40% and RRs ranging from 1.1 to 1.3, PAR estimates for never breastfeeding (compared with breastfeeding for at least 31 months over a lifetime) ranged from 2% to 11% (Figure 4). According to CHIS, 16.4% of white California women aged 40–79 years had never given birth. Therefore, applying the PARs to the California population of parous white women aged 40–79 years, we estimated that never breastfeeding (compared to breastfeeding for 31+ months) could explain between 218 and 1,306 breast cancer cases yearly.

## Discussion

This study quantified the theoretical reductions in breast cancer to be expected from changes in EPRT use, alcohol consumption, physical activity and breastfeeding duration for a range of socioeconomically heterogeneous populations. Because the variation in breast cancer incidence rates, risk factor prevalences, and socioeconomic status across California is generally representative of national variation[11], we believe that the range of PAR estimates we obtained have relevance not only for 8 million white California women, but also could reflect the range across the US white population. Of 13,019 breast cancers diagnosed annually in Californian white women aged 40–79 years, our data suggest that as many as 1,432 (11%) may be attributable to EPRT use, 2,604 (20%) may be attributable to consuming 2 or more alcoholic drinks daily, 1,953 (15%) may be attributable to physical inactivity and 1,432 (11%) may be attributable to never breastfeeding.

Our data suggest that the proportions of women consuming two or more alcoholic drinks daily may have a greater influence on breast cancer incidence than previously estimated, particularly in high-incidence subpopulations. A pooled analysis of 53 studies estimated that 4% of breast cancer cases in developed countries were attributable to alcohol, based on average consumption of 6.0 g/day in controls[4]. Data from the Third National Health and Nutrition Examination Survey, reporting prevalences of light (0.1–6.4 g/day), moderate (6.5–25.9 g/day) and heavy (26+ g/day) drinking of 27%, 13% and 3% respectively, were used to produce a PAR of 2.1% across all levels[10]. Our estimates of possible PARs for consuming two or more drinks daily included a range with much higher values (1% to 20%). At the low end of our RR range, PAR estimates exceeded 5% for Marin and San Francisco counties, which have among the highest breast cancer rates among all counties in California and in the non-California regions of the SEER program[24]. If prevalence of alcohol consumption now is higher than in previously examined populations; a public health effort aimed at reducing alcohol consumption below two drinks daily may be an increasingly important strategy for breast cancer prevention in particular subpopulations of white women. A more specific recommendation needs to consider the balance of health risks and benefits for alcohol consumption, which may protect against ischemic heart disease and myocardial infarction[25], diseases with greater mortality risks than breast cancer. An alternative strategy to reducing alcohol intake[26] may be for women to increase folate intake through supplementation or food supply fortification, as recent evidence from the Nurses Health Study showed no adverse influence of alcohol on breast cancer occurrence among women with high plasma folate[27].

Although the presumed effect of physical activity on breast cancer risk is not large (RR = 1.3–1.4), the highly prevalent nature of physical inactivity corresponded to a California-wide PAR of 7.5%. Especially at the high end of our estimated PAR range (15%), the potential for physical activity interventions to reduce breast cancer incidence may be underestimated. The CHIS statewide average of 27% of women reporting inactivity is higher than that reported in previous PAR estimation efforts[28] and underscores the importance of ongoing public health efforts to increase physical activity for its well-known, wide range of health benefits.

We found that use of EPRT at the levels occurring in 2001 may have explained 2.5% to 5.6% of breast cancer in white California women, but could have been responsible for as much as 11%, depending on the associated RR. Coombs et al. recently used CHIS data to estimate that HT (irrespective of formulation) was attributable for 4.3–17.4% of breast cancer in California in 2001, depending on the RR[9]. Findings from the Women's Health Initiative (WHI) on risks of long-term EPRT use, released in 2002 after the collection of the data used in this analysis, appear to have had a dramatic effect on EPRT prescribing and usage patterns, with declines of up to 50% reported in some cohorts. Thus, long-term use is now much less likely to be recommended by physicians to reduce risks of osteoporosis and cardiovascular disease[29], although some women continue to use EPRT for treatment of menopausal symptoms. Our results would predict that reductions in EPRT use related to the WHI results could result in breast cancer incidence rate declines between 5% and 15%, depending on population characteristics.

Our findings also indicate that the number of breast cancer cases occurring in California might be reduced by as much as 11% by encouraging breastfeeding for 31 months or more (a time period derived from a meta-analysis that could be refined in further analyses). Lengthening the duration of breastfeeding among childbearing women depends in part on individual circumstances, including parity and medical conditions interfering with breastfeeding, as well as on societal influences beyond individual control (e.g., cultural support of breastfeeding, workplace policies for maternal leave and breast pumping). Nevertheless, our data support continued public policy efforts to encourage longer duration of breastfeeding in childbearing women, which for a woman bearing two children equates to breastfeeding each child slightly more than one year, particularly given the overwhelming evidence of breastfeeding benefit to the infant.

The PAR has had many previous applications to breast cancer, predominantly to estimate the percentage of cases "explained" by established risk factors using results from a single study[10,28,30,31]. Madigan et al. estimated that well-established risk factors for breast cancer (later age at first birth, nulliparity, higher income, and family history of breast cancer) together accounted for 41% of U.S. breast cancer cases[30]. Mezzetti et al. estimated that the attributable risk in an Italian population was 10.7% for high alcohol consumption, 15.0% for low beta-carotene intake, and 11.6% for low physical activity, with the three factors together accounting for 33% of breast cancer cases[28]. Rockhill et al. estimated that 25% (95% CI 6% – 48%) of breast cancer cases could be attributed to menarche before age 14 years, nulliparity/first birth after age 20 years, family history, and history of benign breast biopsy[31]. However, these studies did not aim to quantify potential case count reductions from changes in risk factor prevalence, as we have done.

Our study has several limitations. Our statistic of choice, the PAR, is a useful measure for quantifying the extent of disease reduction possible through behavior modification, but its interpretation can be complex, particularly as it relates to the interrelationships of factors of interest with other established risk factors and changes in population prevalence. In addition, the PAR does not incorporate processes of risk latency and reversibility for exposures; for example, breast cancer risk increases with duration of EPRT use and declines after EPRT cessation[21], effects we could not capture. The PAR also is sensitive to the reference (i.e., minimum risk) category chosen. To minimize the effect of these limitations, we restricted our analysis to a relatively homogeneous group, white women aged 40–79 years, and used systematically collected estimates of exposure prevalence in categories for which multiple RR estimates were available. Exposure prevalence estimates, although based on the best available population data, are subject to sampling variation, response and misclassification bias. The AskCHIS program uses a weighting procedure to compensate for differential selection probabilities for households and persons, which attempts to minimize selection bias and adjusts, to the extent possible, for undercoverage in the sampling frames[15]. Although RR estimates were taken from pooled analyses or other multi-study efforts, they remain subject to bias or uncontrolled confounding. To help account for this problem, we examined a range of possible RR values. Empirically derived ranges of values for RR and exposure prevalence may be more difficult to interpret but are more useful for future public health consideration. Lastly, our PAR estimates cannot be summed together to produce a combined PAR estimate for removing all three risk factors from the population, because these factors are likely to be correlated; moreover, we did not calculate multivariate PARs because RRs for the combinations of all three risk factors were not available.

We restricted our analysis to white non-Hispanic women because few studies have included sufficient numbers of women from non-white racial/ethnic groups to obtain reliable RR estimates for the exposures we considered here[32]. Based on the limited data available[32,33], it is likely that incidence reduction in nonwhite groups also may be anticipated from changes in these behaviors. Statewide CHIS data suggested that the prevalences of combined EPRT use and consumption of at least two alcoholic drinks per day were considerably lower in African American, Asian and Hispanic women in 2001 than in white women, at 7%, 13% and 9%; and 2.7%, 0.8% and 0.9%, respectively. Average annual age-adjusted incidence rates of breast cancer were also much lower in these ethnic groups, at 263, 194 and 197 per 100,000 women per year for the period 1998–2002 in California. Thus, for these factors, PARs for non-white women may be considerably lower than those we estimated in white women. On the other hand, prevalence of physical inactivity was notably higher in African American, Asian and Hispanic women (45%, 45%, 57%, respectively), such that PARs and presumable breast cancer preventive benefits could be higher for remediating physical inactivity in non-white women[33] than our estimates for white women.

## Conclusion

Our results suggest that public health interventions to further reduce the proportions of women taking EPRT, drinking two or more alcoholic beverages daily, being physically inactive or not breastfeeding could substantially decrease the numbers of new breast cancer cases and, furthermore, may have a greater impact on breast cancer risks than previously estimated by others[4,10], particularly in high incidence populations where these risk factors tend to be more prevalent. Given the relative feasibility of implementing these changes and the other health benefits already associated with them, public health efforts toward this end are warranted.

## Abbreviations

EPRT estrogen/progestin replacement therapy

PAR population attributable risk

CHIS California Health Interview Survey

HT hormone therapy

WHI Womens' Health Initiative

CI confidence intervals

RR relative risk

## Competing interests

Drs. Clarke and Purdie have consulted for attorneys preparing litigation regarding hormone therapy.

## Authors' contributions

CC conceived of the study, performed statistical analyses and drafted the manuscript. DP performed statistical analyses, and drafted the manuscript. SG participated in the study design and helped to draft the manuscript. All authors read and approved the final manuscript.

## Pre-publication history

The pre-publication history for this paper can be accessed here:



## Figures and Tables

**Table 1:** Average annual population counts, number of invasive breast cancer cases, and incidence rates in California Health Interview Survey-defined California county groups for non-Hispanic white women aged 40–79, 1998–2002.

**California county group**	**Population**	**Cases**	**Rate**^a^
Siskiyou/Lassen/Modoc/Trinity	20,055	55	258.3
Imperial	6,795	21	273.5
Tulare	37,287	112	281.0
Tehama/Colusa/Glenn	17,096	53	284.8
Kern	72,647	215	285.1
Stanislaus	58,641	179	294.6
Mendocino/Lake	29,368	93	295.9
Madera	15,356	48	299.2
San Joaquin	62,562	195	300.0
Humboldt/Del Norte	28,529	88	304.6
San Luis Obispo	46,319	151	306.0
San Bernardino	163,317	512	307.1
Butte	38,390	131	309.7
Monterey/San Benito	46,548	151	309.8
Riverside	194,069	666	309.9
Merced	20,340	67	310.4
Tuolumne/Alpine/Amador/Calaveras/Inyo/Mariposa/Mono	40,654	138	312.3
Shasta	34,222	115	318.1
Yolo	20,875	66	318.6
Santa Cruz	40,217	123	318.7
Sonoma	87,289	283	322.7
El Dorado	33,311	106	322.8
Fresno	76,816	262	323.2
Santa Barbara	54,635	190	324.2
Placer	49,738	165	328.2
Sutter/Yuba	19,714	68	329.6
Nevada/Plumas/Sierra	28,648	100	329.8
San Diego	349,009	1,193	330.0
Kings	10,115	35	333.5
Los Angeles	744,519	2,656	338.5
Solano	42,593	142	338.6
Orange	348,248	1,217	340.2
Ventura	98,544	344	348.3
Sacramento	161,327	581	349.8
Alameda	139,371	490	353.1
Santa Clara	177,331	643	354.9
Contra Costa	136,317	488	355.7
Napa	22,552	87	361.1
San Mateo	90,534	341	362.6
San Francisco	63,036	242	374.4
Marin	54,688	206	374.8

All California	3,781,621	13,019	331.6

SEER		80,072	315.6

^a ^per 100,000/year and age-adjusted to the 2000 U.S. standard.

**Table 2:** Exposure prevalence and population attributable risk (PAR) for breast cancer among white non-Hispanic women ages 40–79 associated with current use of estrogen/progestin-containing hormone therapy, consumption of 2+ alcoholic beverages/day, and physical inactivity in California Health Interview Survey-defined California county groups.

	**Estrogen/progestin hormone therapy**				**Consumption of 2+ alcoholic beverages**				**Physical inactivity**			
**California county group**	**Exposure prevalence**	**PAR for RR = 1.26**	**Rate**^a ^**if exposure removed**	**N cases due to exposure**	**Exposure prevalence**	**PAR for RR = 1.4**	**Rate**^a ^**if exposure removed**	**N cases due to exposure**	**Exposure prevalence**	**PAR for RR = 1.3**	**Rate**^a ^**if exposure removed**	**N cases due to exposure**

Siskiyou/Lassen/Trinity/Modoc	10.00%	2.50%	251.8	1	5.00%	2.00%	253.2	1	30.8	8.50%	236.5	8
Imperial	16.70%	4.20%	262.1	1	0.00%	0.00%	273.5	0	34.1	9.30%	248.1	4
Tulare	19.40%	4.80%	267.5	5	5.60%	2.20%	274.9	2	33	9.00%	255.7	17
Tehama/Glenn/Colusa	11.80%	3.00%	276.4	2	0.00%	0.00%	284.8	0	32.1	8.80%	259.8	9
Kern	11.40%	2.90%	276.9	6	8.60%	3.30%	275.7	7	30.7	8.40%	261.1	25
Stanislaus	13.00%	3.30%	285	6	7.30%	2.80%	286.3	5	34.6	9.40%	266.9	22
Mendocino, Lake	13.30%	3.40%	286	3	6.50%	2.50%	288.5	2	30.5	8.40%	271.1	13
Madera	13.30%	3.40%	289.2	2	0.00%	0.00%	299.2	0	24.8	6.90%	278.5	5
San Joaquin	12.10%	3.10%	290.8	6	4.60%	1.80%	294.6	3	29.4	8.10%	275.7	23
Humboldt/Del Norte	17.90%	4.40%	291.1	4	7.10%	2.80%	296.1	2	27.5	7.60%	281.4	8
San Luis Obispo	15.70%	3.90%	294	6	5.90%	2.30%	299	3	23.5	6.60%	285.8	19
San Bernadino	16.80%	4.20%	294.3	21	5.80%	2.30%	300.2	12	29.8	8.20%	281.9	52
Butte	12.50%	3.20%	300	4	7.50%	2.90%	300.7	4	26.9	7.50%	286.6	21
Monterey/San Benito	18.20%	4.50%	295.8	7	9.10%	3.50%	298.9	5	26.5	7.40%	287	17
Riverside	13.10%	3.30%	299.7	22	7.50%	2.90%	300.8	19	30.2	8.30%	284.2	115
Merced	16.70%	4.20%	297.5	3	0.00%	0.00%	310.4	0	36.7	9.90%	279.6	10
Tuolumne/Calaveras/Amador/Inyo/Mariposa/Mono/Alpine	14.00%	3.50%	301.4	5	11.60%	4.40%	298.4	6	23.7	6.60%	291.6	19
Shasta	14.30%	3.60%	306.7	4	5.70%	2.20%	311	3	26.8	7.40%	294.4	14
Yolo	19.10%	4.70%	303.6	3	9.50%	3.70%	306.9	2	30.8	8.50%	291.7	5
Santa Cruz	21.10%	5.20%	302.2	6	12.80%	4.90%	303.2	6	19.4	5.50%	301.2	2
Sonoma	18.60%	4.60%	307.8	13	12.80%	4.90%	307	14	22.8	6.40%	302	19
El Dorado	13.20%	3.30%	312.1	3	12.80%	4.90%	307.1	5	20.7	5.80%	303.9	5
Fresno	16.90%	4.20%	309.6	11	6.50%	2.50%	315	7	28.7	7.90%	297.6	33
Santa Barbara	16.70%	4.20%	310.7	8	7.40%	2.90%	314.9	5	24.8	6.90%	301.7	25
Placer	13.20%	3.30%	317.3	5	9.40%	3.60%	316.3	6	20.5	5.80%	309.2	11
Sutter/Yuba	10.00%	2.50%	321.3	2	5.00%	2.00%	323.1	1	30.9	8.50%	301.6	9
Nevada/Plumas/Sierra	17.20%	4.30%	315.7	4	13.80%	5.20%	312.6	5	21.5	6.10%	309.8	11
San Diego	19.30%	4.80%	314.2	57	9.90%	3.80%	317.4	46	23.4	6.60%	308.4	117
Kings	11.10%	2.80%	324.1	1	0.00%	0.00%	333.5	0	41.2	11.00%	296.8	5
Los Angeles	18.10%	4.50%	323.3	119	7.90%	3.10%	328.2	81	30.5	8.40%	310.1	347
Solano	15.60%	3.90%	325.4	6	8.90%	3.40%	327	5	23.6	6.60%	316.2	7
Orange	19.30%	4.80%	324	58	9.20%	3.60%	328.1	43	27.7	7.70%	314.1	123
Ventura	17.00%	4.20%	333.6	15	5.00%	1.90%	341.5	7	26.2	7.30%	322.9	26
Sacramento	18.20%	4.50%	334	26	10.70%	4.10%	335.5	24	25.1	7.00%	325.3	56
Alameda	22.90%	5.60%	333.3	27	12.10%	4.60%	336.7	23	24.1	6.70%	329.3	31
Santa Clara	18.80%	4.70%	338.4	30	9.30%	3.60%	342.1	23	26.7	7.40%	328.6	60
Contra Costa	20.10%	5.00%	338	24	11.00%	4.20%	340.8	20	24.2	6.80%	331.6	36
Napa	22.70%	5.60%	341	5	9.10%	3.50%	348.4	3	24.1	6.70%	336.8	11
San Mateo	13.30%	3.40%	350.5	11	14.10%	5.40%	343.2	18	25	7.00%	337.3	36
San Francisco	20.30%	5.00%	355.7	12	13.50%	5.10%	355.2	12	22.2	6.20%	351	21
Marin	20.40%	5.00%	356	10	16.40%	6.10%	351.8	13	16.2	4.60%	357.4	11

All California	17.50%	4.40%	317.2	567	9.00%	3.50%	320.1	450	27.1	7.50%	306.7	1422

^a ^per 100,000/year and age-adjusted to the 2000 U.S. standard

## References

[B1] Kelsey JL, Gammon MD, John EM (1993). Reproductive factors and breast cancer. Epidemiol Rev.

[B2] Hulka BS, Brinton LA (1995). Hormones and breast and endometrial cancers: preventive strategies and future research. Environ Health Perspect.

[B3] Rossouw JE, Anderson GL, Prentice RL, LaCroix AZ, Kooperberg C, Stefanick ML, Jackson RD, Beresford SA, Howard BV, Johnson KC, Kotchen JM, Ockene J (2002). Risks and benefits of estrogen plus progestin in healthy postmenopausal women: principal results From the Women's Health Initiative randomized controlled trial. JAMA.

[B4] Hamajima N, Hirose K, Tajima K, Rohan T, Calle EE, Heath CWJ, Coates RJ, Liff JM, Talamini R, Chantarakul N, Koetsawang S, Rachawat D, Morabia A, Schuman L, Stewart W, Szklo M, Bain C, Schofield F, Siskind V, Band P, Coldman AJ, Gallagher RP, Hislop TG, Yang P, Kolonel LM, Nomura AM, Hu J, Johnson KC, Mao Y, De Sanjose S, Lee N, Marchbanks P, Ory HW, Peterson HB, Wilson HG, Wingo PA, Ebeling K, Kunde D, Nishan P, Hopper JL, Colditz G, Gajalanski V, Martin N, Pardthaisong T, Silpisornkosol S, Theetranont C, Boosiri B, Chutivongse S, Jimakorn P, Virutamasen P, Wongsrichanalai C, Ewertz M, Adami HO, Bergkvist L, Magnusson C, Persson I, Chang-Claude J, Paul C, Skegg DC, Spears GF, Boyle P, Evstifeeva T, Daling JR, Hutchinson WB, Malone K, Noonan EA, Stanford JL, Thomas DB, Weiss NS, White E, Andrieu N, Bremond A, Clavel F, Gairard B, Lansac J, Piana L, Renaud R, Izquierdo A, Viladiu P, Cuevas HR, Ontiveros P, Palet A, Salazar SB, Aristizabel N, Cuadros A, Tryggvadottir L, Tulinius H, Bachelot A, Le MG, Peto J, Franceschi S, Lubin F, Modan B, Ron E, Wax Y, Friedman GD, Hiatt RA, Levi F, Bishop T, Kosmelj K, Primic-Zakelj M, Ravnihar B, Stare J, Beeson WL, Fraser G, Bullbrook RD, Cuzick J, Duffy SW, Fentiman IS, Hayward JL, Wang DY, McMichael AJ, McPherson K, Hanson RL, Leske MC, Mahoney MC, Nasca PC, Varma AO, Weinstein AL, Moller TR, Olsson H, Ranstam J, Goldbohm RA, van den Brandt PA, Apelo RA, Baens J, de la Cruz JR, Javier B, Lacaya LB, Ngelangel CA, La Vecchia C, Negri E, Marubini E, Ferraroni M, Gerber M, Richardson S, Segala C, Gatei D, Kenya P, Kungu A, Mati JG, Brinton LA, Hoover R, Schairer C, Spirtas R, Lee HP, Rookus MA, van Leeuwen FE, Schoenberg JA, McCredie M, Gammon MD, Clarke EA, Jones L, Neil A, Vessey M, Yeates D, Appleby P, Banks E, Beral V, Bull D, Crossley B, Goodill A, Green J, Hermon C, Key T, Langston N, Lewis C, Reeves G, Collins R, Doll R, Peto R, Mabuchi K, Preston D, Hannaford P, Kay C, Rosero-Bixby L, Gao YT, Jin F, Yuan JM, Wei HY, Yun T, Zhiheng C, Berry G, Cooper Booth J, Jelihovsky T, MacLennan R, Shearman R, Wang QS, Baines CJ, Miller AB, Wall C, Lund E, Stalsberg H, Shu XO, Zheng W, Katsouyanni K, Trichopoulou A, Trichopoulos D, Dabancens A, Martinez L, Molina R, Salas O, Alexander FE, Anderson K, Folsom AR, Hulka BS, Bernstein L, Enger S, Haile RW, Paganini-Hill A, Pike MC, Ross RK, Ursin G, Yu MC, Longnecker MP, Newcomb P, Kalache A, Farley TM, Holck S, Meirik O (2002). Alcohol, tobacco and breast cancer - collaborative reanalysis of individual data from 53 epidemiological studies, including 58,515 women with breast cancer and 95,067 women without the disease. Br J Cancer.

[B5] Collaborative Group on Hormonal Factors in Breast Cancer (2002). Breast cancer and breastfeeding: collaborative reanalysis of individual data from 47 epidemiological studies in 30 countries, including 50302 women with breast cancer and 96973 women without the disease. Lancet.

[B6] Friedenreich CM (2001). Physical activity and cancer prevention: from observational to intervention research. Cancer Epidemiol Biomarkers Prev.

[B7] (2002). Physical activity across the cancer continuum: report of a workshop: review of existing knowledge and innovative designs for future research. Cancer.

[B8] Coombs NJ, Taylor R, Wilcken N, Boyages J (2005). HRT and breast cancer: impact on population risk and incidence. Eur J Cancer.

[B9] Coombs NJ, Taylor R, Wilcken N, Fiorica J, Boyages J (2005). Hormone replacement therapy and breast cancer risk in California. Breast J.

[B10] Tseng M, Weinberg CR, Umbach DM, Longnecker MP (1999). Calculation of population attributable risk for alcohol and breast cancer (United States). Cancer Causes Control.

[B11] Reynolds P, Hurley S, Goldberg DE, Anton-Culver H, Bernstein L, Deapen D, Horn-Ross PL, Peel D, Pinder R, Ross RK, West D, Wright WE, Ziogas A (2004). Regional variations in breast cancer among California teachers. Epidemiology.

[B12] Breslow NE, Day NE (1980). Methods in Cancer Research Vol. 1: The Analysis of Case-Control Studies.

[B13] US Census Bureau Census 2000 Summary File 3, Matrices P19, P36, P37, P38, PCT24, and PCT25.

[B14] National Cancer Institute DCCPSSRPCSB (2005). Surveillance, Epidemiology, and End Results (SEER) Program (www.seer.cancer.gov) SEER*Stat Database: Incidence - SEER 9 Regs Public-Use, Nov 2004 Sub (1973-2002.

[B15] California Health Interview Survey (2002). CHIS 2001 Methodology Series: Report 4 – Response Rates..

[B16] California Health Interview Survey (2004). CHIS 2001 Adult Public Use File, Release 3 [computer file].

[B17] Grady D, Gebretsadik T, Kerlikowske K, Ernster V, Petitti D (1995). Hormone replacement therapy and endometrial cancer risk: a meta-analysis. Obstet Gynecol.

[B18] Newton ER (2004). The epidemiology of breastfeeding. Clin Obstet Gynecol.

[B19] Nelson HD, Humphrey LL, Nygren P, Teutsch SM, Allan JD (2002). Postmenopausal hormone replacement therapy: scientific review. JAMA.

[B20] Chen WY, Colditz GA, Rosner B, Hankinson SE, Hunter DJ, Manson JE, Stampfer MJ, Willett WC, Speizer FE (2002). Use of postmenopausal hormones, alcohol, and risk for invasive breast cancer. Ann Intern Med.

[B21] Collins JA, Blake JM, Crosignani PG (2005). Breast cancer risk with postmenopausal hormonal treatment. Hum Reprod Update.

[B22] Smith-Warner SA, Spiegelman D, Yaun SS, van den Brandt PA, Folsom AR, Goldbohm RA, Graham S, Holmberg L, Howe GR, Marshall JR, Miller AB, Potter JD, Speizer FE, Willett WC, Wolk A, Hunter DJ (1998). Alcohol and breast cancer in women: a pooled analysis of cohort studies. JAMA.

[B23] Wrensch M, Chew T, Farren G, Barlow J, Belli F, Clarke C, Erdmann CA, Lee M, Moghadassi M, Peskin-Mentzer R, Quesenberry CPJ, Souders-Mason V, Spence L, Suzuki M, Gould M (2003). Risk factors for breast cancer in a population with high incidence rates. Breast Cancer Res.

[B24] Clarke CA, Glaser SL, West DW, Ereman RR, Erdmann CA, Barlow JM, Wrensch MR (2002). Breast cancer incidence and mortality trends in an affluent population: Marin County, California, USA, 1990-1999. Breast Cancer Res.

[B25] Rimm EB, Klatsky A, Grobbee D, Stampfer MJ (1996). Review of moderate alcohol consumption and reduced risk of coronary heart disease: is the effect due to beer, wine, or spirits. Bmj.

[B26] Colditz GA (2005). Epidemiology and prevention of breast cancer. Cancer Epidemiol Biomarkers Prev.

[B27] Zhang SM, Willett WC, Selhub J, Hunter DJ, Giovannucci EL, Holmes MD, Colditz GA, Hankinson SE (2003). Plasma folate, vitamin B6, vitamin B12, homocysteine, and risk of breast cancer. J Natl Cancer Inst.

[B28] Mezzetti M, La Vecchia C, Decarli A, Boyle P, Talamini R, Franceschi S (1998). Population attributable risk for breast cancer: diet, nutrition, and physical exercise. J Natl Cancer Inst.

[B29] Vogel RA (2003). The changing view of hormone replacement therapy. Rev Cardiovasc Med.

[B30] Madigan MP, Ziegler RG, Benichou J, Byrne C, Hoover RN (1995). Proportion of breast cancer cases in the United States explained by well-established risk factors. J Natl Cancer Inst.

[B31] Rockhill B, Weinberg CR, Newman B (1998). Population attributable fraction estimation for established breast cancer risk factors: considering the issues of high prevalence and unmodifiability. Am J Epidemiol.

[B32] Bernstein L, Teal CR, Joslyn S, Wilson J (2003). Ethnicity-related variation in breast cancer risk factors. Cancer.

[B33] John EM, Horn-Ross PL, Koo J (2003). Lifetime physical activity and breast cancer risk in a multiethnic population: the San Francisco Bay area breast cancer study. Cancer Epidemiol Biomarkers Prev.

